# The Effect of ICA and Non-negative Matrix Factorization Analysis for EMG Signals Recorded From Multi-Channel EMG Sensors

**DOI:** 10.3389/fnins.2020.600804

**Published:** 2020-12-01

**Authors:** Yeongdae Kim, Sorawit Stapornchaisit, Makoto Miyakoshi, Natsue Yoshimura, Yasuharu Koike

**Affiliations:** ^1^Department of Information and Communications Engineering, Tokyo Institute of Technology, Meguro, Japan; ^2^Swartz Center for Computational Neuroscience, Institute for Neural Computation, University of California, San Diego, San Diego, CA, United States; ^3^Institute of Innovative Research, Tokyo Institute of Technology, Yokohama, Japan; ^4^PRESTO, Japan Science and Technology Agency (JST), Tokyo, Japan; ^5^ATR Brain Information Communication Research Laboratory Group, Kyoto, Japan; ^6^Integrative Brain Imaging Center, National Center of Neurology and Psychiatry, Tokyo, Japan

**Keywords:** finger movement, electromyography, muscle synergy, independent component analysis, deep muscle

## Abstract

Surface electromyography (EMG) measurements are affected by various noises such as power source and movement artifacts and adjacent muscle activities. Hardware solutions have been found that use multi-channel EMG signal to attenuate noise signals related to sensor positions. However, studies addressing the overcoming of crosstalk from EMG and the division of overlaid superficial and deep muscles are scarce. In this study, two signal decompositions—independent component analysis and non-negative matrix factorization—were used to create a low-dimensional input signal that divides noise, surface muscles, and deep muscles and utilizes them for movement classification based on direction. In the case of index finger movement, it was confirmed that the proposed decomposition method improved the classification performance with the least input dimensions. These results suggest a new method to analyze more dexterous movements of the hand by separating superficial and deep muscles in the future using multi-channel EMG signals.

## Introduction

Electromyography (EMG) measures the electrical impulses from the muscle contraction induced by the central nervous system for voluntary body movement. The surface EMG signal contains different muscle signals and various noises such as baseline noise and movement artifacts (De Luca et al., 2010). These noises and crosstalk between muscles can misguide EMG analysis leading to erroneous interpretation; hence, there are various studies that focus on attenuating undesirable signals (De Luca et al., 2010). However, it is still challenging to detect single muscle activity from EMG (Schieber, 1995; Keen and Fuglevand, 2004). Gazzoni et al., 2014 applied non-negative matrix factorization (NMF) to multi-channel EMG signals and distinguished the position on each forearm per movement of the wrist and single finger joint. Their study investigated deep muscle activities under singular joint movement and confirmed the feasibility of multi-channel EMG signals-based muscles synergy so as to identify deep muscle region. However, they did not dig deep into the dexterous finger movement and nor the structure of muscle synergy per joint movement. Finger movement of high dexterity can be achieved through multiple muscle movements; however, this causes significant crosstalk in the forearm EMG measurements. Thereby, additional steps need to be taken in dexterous finger movements estimation using multi-channel EMG signal.

Independent component analysis (ICA) is a general-purpose statistical technique that can linearly transform random data into independent components (ICs) (Hyvärinen and Oja, 2000). Hence, it is often employed in biomedical signal processing, especially for EEG, where noise and various movement artifacts are removed through ICA before signal analysis (Jung et al., 2000). In EMG analysis, it was confirmed that ICA reduces the root mean squared error of the monopolar EMG signals that measure muscle force (Staudenmann et al., 2007).

Muscle synergy originated from the idea that the brain would not control individual muscles and commands a high degree of complexity to control our daily movements (d’Avella et al., 2003). In addition, the NMF algorithm by Lee and Seung (2001) has established a standard method for muscle synergy calculation. Since then several EMG studies have proposed low-dimensional input-based myoelectric models using muscle synergy. Of these, the fast type NMF computation algorithm (Cichocki and Phan, 2009) was used to calculate muscle synergy in this study.

This study tests how well multichannel EMG signals can estimate the direction of index finger movements using two signal decomposition ICA and NMF. We examined the following three parameters to compare EMG-based synergy (EMG-synergy) and ICs-based synergy (ICA-synergy): (1) robustness of the synergy structure calculation in the two elbow posture, (2) preferred direction compared with anatomical basis, and (3) classification performance on the eight direction finger movements using convolutional neural network (CNN).

## Materials and Methods

Experimental data were obtained from the study of Yoshimura et al. (2017). The data were taken from six right-handed healthy participants (two females and four males, aged 40.67 ± 7.23). The research protocol was approved by the University of California San Diego Ethics Committee (approval number 14353) and was conducted in accordance with the Helsinki Declaration. Written consent was obtained from each participant prior to the experiment (Yoshimura et al., 2017). This study focused on 96 channel EMG signals acquired from Biosemi active Two amplifier system with active sensors (Biosemi, Amsterdam, Netherlands), and its analysis with respect to cursor movement directions. The EMG signals were sampled at 2,048 Hz and Cursor movement at 100 Hz.

**FIGURE 1:**
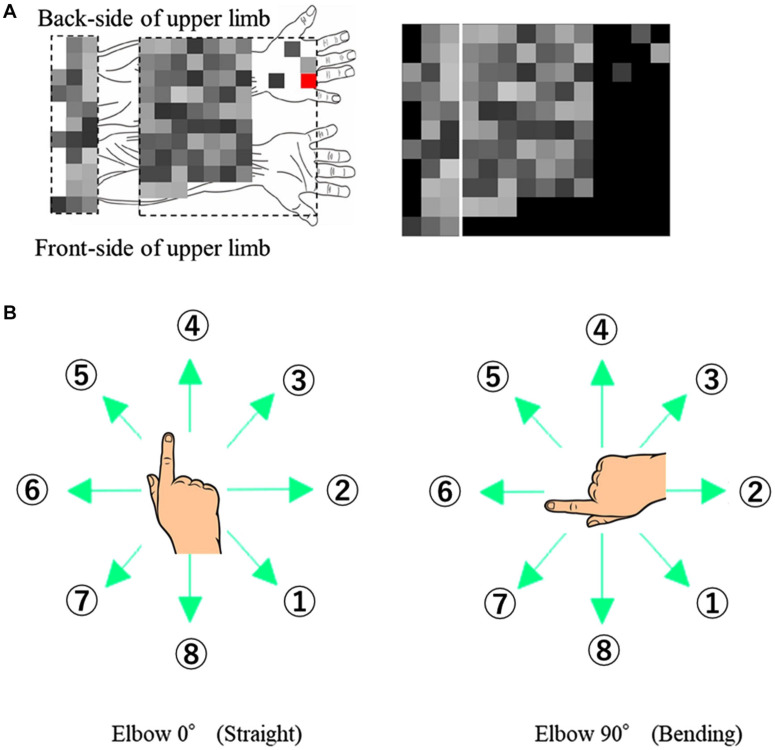
Experimental Design. **(A)** EMG signals with respective channel positions on the upper limb. The red channel on the back of the hand is used as the ground reference and not for ICA or muscle synergy. **(B)** Eight target directions for the two different elbow angles (0° and 90°).

Before the EMG acquisition, the coordinate positions of the EMG sensors and the hand and elbow joint were measured. The coordinate positions, measured using a posture functional capacity evaluation system (zebris Medical GmbH, Isny, Germany) and the placement of 96 channel-EMG electrodes are shown in Figure 1A.

The participants made finger movements by shifting the cursor in eight different directions, at two elbow angles (0° and 90°) as shown in Figure 1B. The numbering of directions per posture was constant to extract the extrinsic coordinate reaction. The participants performed 80-time repetitions on each posture and direction.

### EMG Preprocessing

The entire EMG data were loaded into MATLAB and digitally filtered by notch and bandpass filters of 50 and 10–1,000 Hz respectively. The ground reference was fixed as the least vibrating point based on standard deviation parameters (all participants had the same measurement points on the hand). Therefore only 95 channels were considered for evaluation.

### ICA and NMF Signal Processing

**FIGURE 2:**
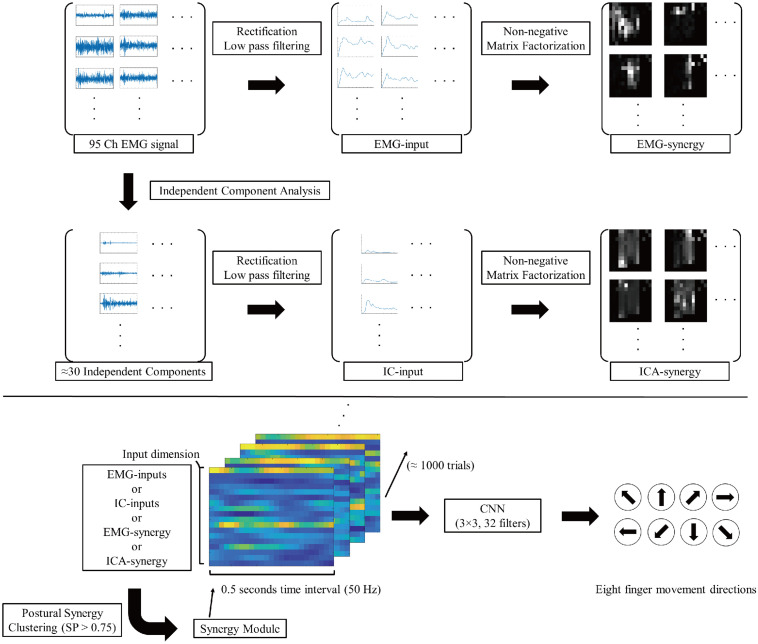
Signal flow diagram of EMG signals. The 95ch preprocessed EMG signal convert to four different type of inputs (EMG-input, IC-input, EMG-synergy, and ICA-synergy) and applied in CNN for eight finger movement direction classification.

Figure 2 shows the signal flow of EMG signals with respective processing after such preprocessing. Similar to the case of Yoshimura et al. (2017), the adaptive mixture independent component analysis (AMICA)^1^ from EEGLAB (Delorme and Makeig, 2004) was used as an algorithm for ICA.

Independent component analysis is one of the representative blind source separation method for cocktail party problem like condition. In this study, multi-channel EMG signals are mixed with mutually different muscle activity signals with numerous noises. Thereby, ICA analysis is demanding to derive deep muscle activities. The basic method of ICA is deriving:

X=AS.

Where *X* is C by N matrix of EMG sensor signals with C is the number of input channels and N is time points; *S* is C by N matrix of the independent source signals which are hypothesized mutually independent, and A is C by C mixing matrix showing how the source signal S is composed in each sensor. ICA derive the basic vectors in the way minimizing the mutual information and maximizing the non-Gaussianity. Each trial had a duration of 2 s, which is 2 s of the post-onset recording after target onset. Among outputs from AMICA, ICs that had white noise characteristics on the trials were excluded from further analysis. The ICA analysis of the 95 channel EMG signals on 1,280 trials from the six participants yielded between 22 to 36 ICs depending on the participants [mean: 29.8, standard deviation (SD): 5.1].

Muscle synergy derivation was also conducted using output from AMICA followed by additional preprocessing. The signals were normalized with mean activation among the whole movements (Halaki and Ginn, 2012), then rectified and filtered using a second-order Butterworth low-pass filter with 5 Hz cutoff frequency, to make a pseudo-joint-torque parameter from EMG signals (Koike and Kawato, 1995). The muscle synergy computation was performed on two different input signals, EMG signals, and ICs after filtering them into pseudo-joint-torque parameters. As a signal decomposition tool for synergy derivation, the HALS method was used (Cichocki and Phan, 2009). That is,

E=TM.

Where *E* is a m by n matrix of EMG signals or selective ICs with n is the number of sensors or ICs and m is time points; T =[t_1_,…,t_s_] is the time series activation coefficient of the synergies in m by s matrix where s is the number of synergies; M = [e_1_,…,e_s_]^T^ is the muscle synergy set in s by n matrix, and e_1_ = [c_1_,…,c_n_] represent a single vector of muscle synergy. Each *T* and *M* are computed iteratively under the rule of minimizing the Euclidean difference between original signal (*E*) and reconstructed signal (*TM*) while having non-negativity.

**FIGURE 3:**
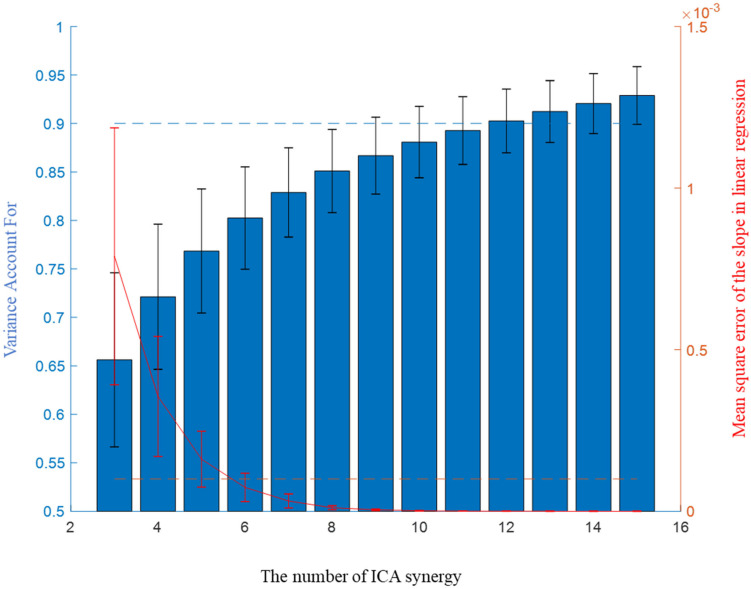
Mean Variance Account For (VAF) and slope of VAF of ICA-synergy in the six participants.

Three to fifteen ICA-synergy sets and 3–30 EMG-synergy were computed per participant. The smallest number, each different per participant, that satisfied the following two conditions was selected as the number of muscle synergy: more than 0.9 Variance Account For (VAF) which was commonly used in other studies (Cheung et al., 2005); Clark et al., 2010; Santuz et al., 2017) and less than 10^−4^ mean squared error of the linear regression of VAF per muscle synergy number (d’Avella et al., 2006). Figure 3 shows two condition parameters with the number of ICA-synergy in six participants. From these conditions, 10–26 EMG-synergy (mean: 18.2, SD: 5.5) and 9–18 ICA-synergy (mean: 12.2, SD: 3.2) were derived from the whole dataset and used depending on the participants throughout the study. The same number of synergies was used when using posture dependent synergy to see the consistency of the synergy structure.

### Muscle Synergy Modulization From Postures

To investigate the difference of muscle synergies depending on the posture, muscle synergies were derived from three different conditional data (Elbow 0°, Elbow 90°, and Total dataset). The synergies from the three conditions were clustered and grouped similar structures depending on the mutual scalar product (SP) (Cheung et al., 2012; Matsunaga et al., 2017) using the Unweighted Pair-Group Method with Arithmetic mean (Nei and Kumar, 2000). Muscle synergies, having SP > 0.75, were assumed to form the same synergy module. The preferred direction was calculated using cosine tuning using the mean amplitude of muscle (or synergy) signal m(θ) in eight direction θ with a linear regression m(θ) = a_0_a_1_ *cos*⁡(θ) + a_2_ *sin*⁡(θ) (Gentner et al., 2013). Then the resultant preferred directions are compared to see the consistency of the signal in two elbow postures. The preferred directions of the synergy in Elbow 90 were compensated by shifting 90° clockwise. In cosine tuning linear regression under normalized form, cosine tuning weight is defined as W = $\sqrt{{\text{a}}_{1}^{2}+{\text{a}}_{2}^{2}}$ that shows the ratio of muscle activation for target finger movements.

### The Classification Model and Truncation of Input Data per Trial

For classification, the input signal was down-sampled to 50 Hz. In the case of EMG, it is difficult to grasp a precise onset due to noise, so the data was sorted based on the cursor movements. The timing of cursor moving more than 2% of movement compared to the final position was set to onset. Based on the cursor onset, 0.5-s data (25 time-points) from −0.2 to 0.3 s was used for each trial. The classification was conducted using four different types of muscle activity signals: EMG-input, IC-input, EMG-synergy, and ICA-synergy. Twenty-five-time points of each signal were used to classify the eight directional finger movements using CNN (Krizhevsky et al., 2012). Multi-channel muscle activities with time interval per trial was implemented as input signals for CNN, and 3 by 3 32 filters were applied for feature extraction. The classification was conducted in fivefold cross-validation on to the whole datasets dividing each elbow posture individually.

## Results

**FIGURE 4:**
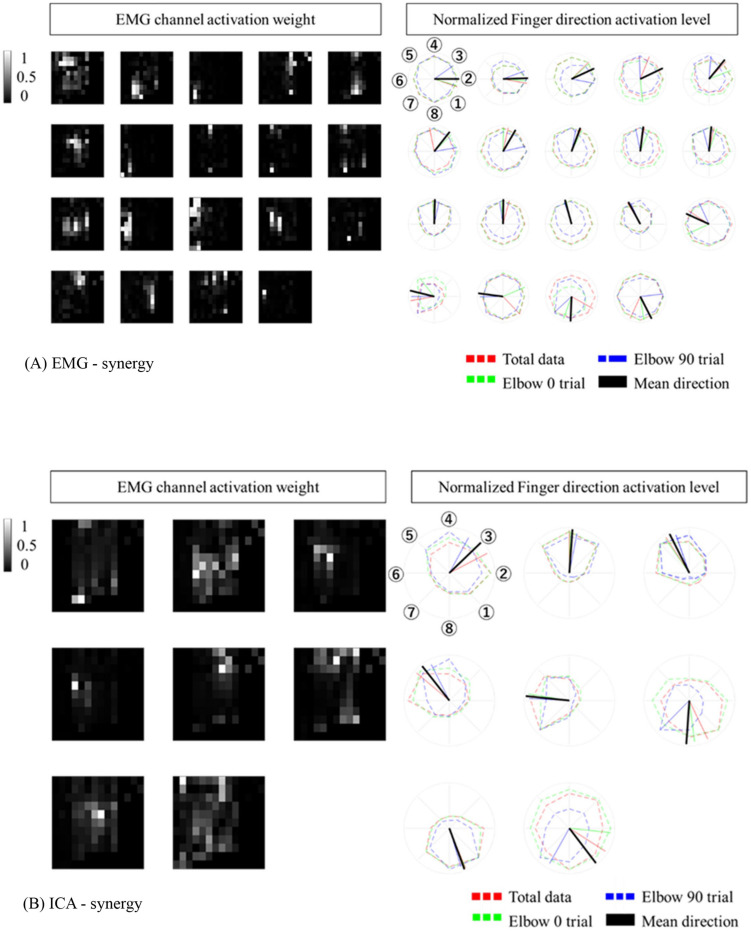
Synergy Modules with corresponding preferred directions. The preferred direction was expressed in different colors according to the trial. Red represents total data, Green represents Elbow 0 trial, Blue represents Elbow 90 trial. **(A)** EMG-synergy Modules with preferred directions. **(B)** ICA-synergy Modules with preferred directions.

When modulating synergies from different elbow posture conditions, the number of EMG-synergy modules is in proportion to the EMG-synergy number (e.g., the ratio between module and synergy is 14.4:18.2 in six participants), and ICA-synergy modules were selected between eight and ten independently to the ICA-synergy number (9–18). Figure 4 shows the EMG channel activations between EMG-synergy and ICA-synergy modules from an individual participant. Between EMG-synergy (Figure 4A) and ICA-synergy (Figure 4B) modules, some EMG channel activation are similar with SP > 0.75, but their inclination for each finger movement is significantly different. Cosine tuning weight W within ICA-synergy is bigger than that of EMG-synergy module (*p* < 0.0001, W_ICA = 0.34 ± 0.11, W_EMG = 0.18 ± 0.12, and *n* = 35). As in Figure 4A, most EMG-synergy modules do not show any inclined activation toward specific finger movement direction and even some pointed to different preferred directions per posture. The average preferred direction error within EMG-synergy module is 24.4 ± 25.8°. As in Figure 4B, ICA-synergy modules are more likely to indicate one clear direction than responding in all directions so that the average preferred direction error within the modules is 13.4 ± 18.1°. In both cases, the standard deviation was higher than the mean. Nevertheless, the coherence of the preferred direction differed statistically significant between the two cases for the *t*-test (*p* = 0.0069, n_EMG = 87, and n_ICA = 54).

**FIGURE 5:**
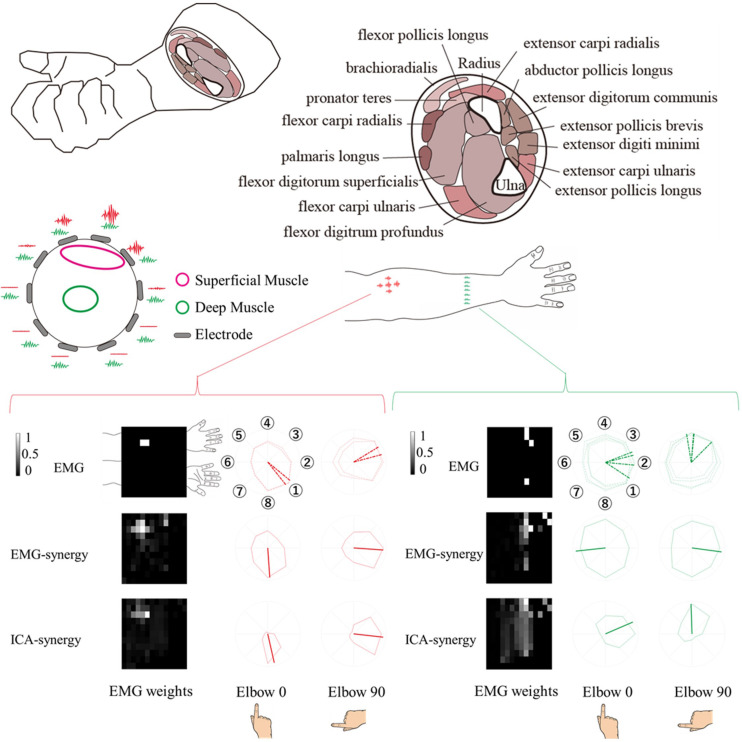
Comparison of horizontal (alongside of the forearm) and vertical (cross-sectional side of the forearm) structure synergy in participants. Based on the physical placement of the muscles, horizontal series of synergy was assumed to be a superficial muscle activity and vertical series a deep muscle activity. The horizontal structure synergy (Red) had equivalent preferred direction both in EMG-synergy and ICA-synergy computation while vertical structure synergy (Green) had distinctive preferred direction change between EMG-synergy and ICA-synergy.

The EMG-synergy and ICA-synergy modules are roughly categorized in two different types, i.e., (i) parallel type wherein the EMG activations are structured alongside the forearm, and (ii) local type wherein the EMG activations are structured in the cross-sectional region of the forearm. Figure 5 shows the comparison between the local and parallel muscles synergies in EMG-synergy and ICA-synergy with corresponding EMG channel activations. For parallel types, both EMG-synergy and ICA-synergy with corresponding EMG channel activations have similar preferred direction toward specific finger movement direction, which is the flexion of the index finger. In local type, EMG-synergy causes an inconsistent preferred direction between Elbow 0 and Elbow 90 conditions on the basis of anatomical point of view while EMG has anatomically consistent activation in two elbow postures. ICA-synergy in this type points to the similar movement direction with corresponding EMG activations, but a clearer preferred direction toward the ulnar abduction of the index finger.

**FIGURE 6:**
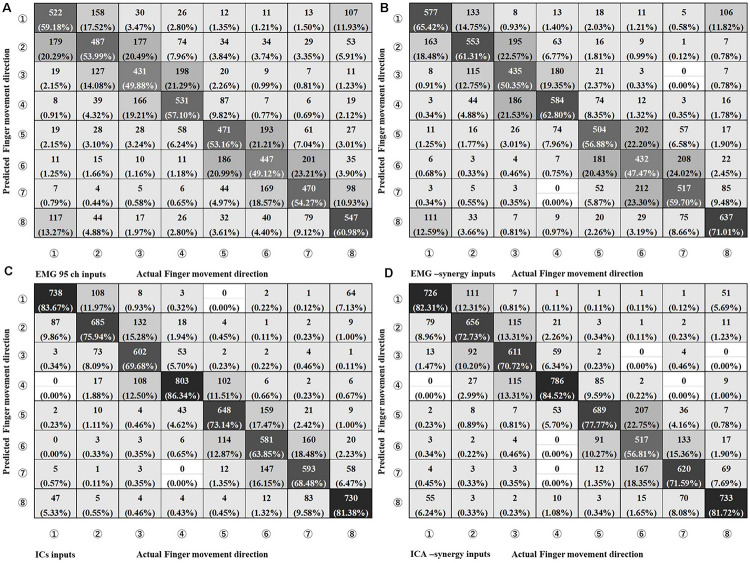
Confusion Matrix of four different input types on Intrinsic Coordinate eight finger movements estimation. **(A)** EMG 95ch inputs, **(B)** EMG-synergy inputs (mean: 18.2, SD: 5.5), **(C)** ICs inputs [mean: 29.8, standard deviation (SD): 5.1], and **(D)** ICA-synergy inputs (mean: 12.2, SD: 3.2).

In the CNN classification of each individual input (EMG-input, IC-input, EMG-synergy, and ICA-synergy) per coordinate (i.e., extrinsic and intrinsic coordinates), classification performances of the intrinsic coordinate are higher than those of the extrinsic coordinate for all inputs. (Extrinsic vs. Intrinsic: p_EMG-input < 0.0001, p_IC-input = 0.0013, p_EMG-synergy = 0.0034, p_ICA-synergy = 0.011, and *n* = 30). Therefore, the estimations of the eight movements of the index finger are compared under intrinsic coordinate and are shown in Figure 6 as confusion matrices. The results show that there is no statistical difference between EMG-input vs. EMG-synergy and ICA-input vs. ICA-synergy (EMG-input vs. EMG-synergy, *p* = 0.18, IC-input vs. ICA-synergy, *p* = 0.86, *n* = 30), and the differences between EMG-input vs. IC-input and EMG-synergy vs. ICA-synergy are significant (EMG-input vs. IC-input, *p* < 0.0001, EMG-synergy vs. ICA-synergy, *p* < 0.0001, *n* = 30). When the modules from two elbow postures are applied as another synergy, the intrinsic classification performance decreased a bit but there was no statistical difference when compared with the classification performance from the synergies derived from the whole data (EMG-synergy vs. EMG-synergy module, *p* = 0.35, ICA-synergy vs. ICA-synergy module, *p* = 0.20, *n* = 30).

## Discussion

The primary goal of this study is to elucidate the effects of ICA and NMF in muscle identification from finger movement. In finger movement, both superficial and deep muscles activate simultaneously. Therefore, recognition of deep muscle and their response is necessary. We used preferred direction calculation and finger movement direction decoding under two postures to evaluate the effect between with and without ICA and NMF through comparisons.

As for the EMG-synergy modules, they show robustness EMG channel activation region across the elbow postures which is similar with those of the ICA-synergy. However, these characteristics do not improve finger movement direction classification performance. Moreover, the EMG-synergy modules activation per finger movement direction showed that NMF computation merely grouped the EMG signal co-activation without any noise reduction. Thereby, the EMG-synergy may be contaminated by noise or noise itself.

On the other hand, the ICA-synergy activation per finger movement direction showed that ICA computation magnified the inclination of synergy structure toward the preferred direction. This result is similar to findings of the past research wherein noise in muscle force estimation was reduced through ICA (Staudenmann et al., 2007). ICA clearly discriminate the muscle crosstalk on the same channel and more importantly, set apart the noise component signals.

The experiment conducted index finger-based cursor movements in eight directions resulting in both deep and superficial muscle coactivation. Due to physical constraints, it is not possible to measure the actual activity of superficial and deep muscles and the resulting muscle signals simultaneously. However, as shown in the left-up side of Figure 5, in the case of superficial muscle activity, the myoelectric signal from the nearby skin surface would be large and the signal is not well received in other areas. On the premise that the signal from deep muscle activity would be evenly captured throughout the cross-sectional region of the muscle. Thereby the parallel and local type of EMG-synergy and ICA-synergy modules were designated to be superficial and deep muscle activity as follows. The superficial muscle modules have a high signal-noise ratio so that the preferred direction has a distinct inclination toward the index finger flexor as shown in Figure 5. However, multichannel EMG sensors collect mixed signals from several muscle co-activities with a multi-layer structure on the forearm muscles (Blanc and Dimanico, 2010). with the crosstalk and other heterogeneous noise in EMG after preprocessing, the preferred directions from each sensor become vague and such trends could hide deep muscle signals. In Figure 5, the cross-sectional EMG-synergy module and corresponding EMG inputs that are regarded as deep muscle activities do not incline to any of eight directions, and only ICA-synergy inclined to ulnar abduction of the index finger. Here, ICA performed sensor configuration (Staudenmann et al., 2006) or replacement of temporal whitening (Clancy and Hogan, 1995), separating noise and artifacts of multiple EMG signals and even dividing muscle activities, maximizing their dimensionality to input signal dimension. Muscle synergy computation on the selective ICs reassembled the same muscle activation by adjusting their dimensionality in minimized form. Thus, ICA-synergy succeeded in discriminating the deep and superficial muscles that led to higher classification performance with fewer input signal synergies than other control inputs. Therefore, clear preferred direction and improved classification performance of ICA-synergy represent the necessity of ICA before muscle synergy computation, which even holds the minimum number of input channels.

The role of ICA and NMF is much clearer in CNN classification performance. When each performance before and after each blind source separation was compared, ICA drastically improved the classification performance while NMF minimized the number of input signals. 95 channel EMG signal lessen to 18.2 ± 5.5 inputs, and 29.8 ± 5.1 ICs to 12.2 ± 3.2. In our previous study that saw multiple joints movement (Kim et al., 2020), specifically complex wrist joint and grasping regression, muscle synergy took the role of identifying the primitive of EMG coactivation. In the current study, in the absence of movement intention separation, NMF was merely used to minimize the number of inputs because such pre-separation is not required.

In the study, we succeeded in identifying both superficial and deep muscle signals through sequential ICA and NMF calculation. However, it is necessary to verify whether the calculations could even divide a mixture of superficial and deep muscle signals under more complex movement including multiple fingers and wrist movement coactivation. In addition, the current estimations only progressed up to the classification stage. The state-of-the-art studies on the wrist or upper joint movements mainly focused on the trajectory (Xia et al., 2018) and the joint angle estimation (Kawase et al., 2017) at a continuous level which is demanding for the prosthesis users (Biddiss, 2009). Thereby, further research is still needed to relate and examine the feasibility of this current ICA-synergy input to continuous estimation models.

## Data Availability Statement

The data analyzed in this study is subject to the following licenses/restrictions: The datasets analyzed in this manuscript are not publicly available. Requests to access these datasets should be directed to YKi, kim.y.ah@m.titech.ac.jp.

## Ethics Statement

The studies involving human participants were reviewed and approved by the research protocol was approved by the University of California San Diego Ethics Committee (approval number 14353) and were conducted in accordance with the Helsinki Declaration. The patients/participants provided their written informed consent to participate in this study.

## Author Contributions

NY and YKo developed the concept. SS, YKo, MM, and NY developed the experimental design. SS, YKi, MM, NY, and YKo collected the data. YKi, NY, and YKo analyzed the data. YKi and YKo wrote the manuscript. All authors contributed to the article and approved the submitted version.

## Conflict of Interest

The authors declare that the research was conducted in the absence of any commercial or financial relationships that could be construed as a potential conflict of interest.

## Footnotes

1 http://sccn.ucsd.edu/~jason/amica_web.html
